# Inflammatory Bowel Disease in Asia: A Second Chance at Uncovering Environmental Factors

**DOI:** 10.1289/ehp.124-A49

**Published:** 2016-03-01

**Authors:** Lindsey Konkel

**Affiliations:** Lindsey Konkel is a New Jersey–based journalist who reports on science, health, and the environment.

When Siew Ng left Malaysia in the early 1990s to study medicine and gastroenterology in London, inflammatory bowel disease (IBD) was practically nonexistent in her native state of Penang and across much of Asia. But on a single day in the fall of 2015, she saw four patients newly diagnosed with Crohn’s disease at the Prince of Wales Hospital in Hong Kong. “It seems that patients with IBD are dropping from the sky,” says Ng, now a gastroenterologist at The Chinese University of Hong Kong.

Thirty years ago, fewer than 1 in 1 million people in Hong Kong had IBD^1^—the blanket term physicians use to refer to Crohn’s disease and a related condition called ulcerative colitis. Both of these immune-mediated diseases lead to chronic gut inflammation that often results in surgery (irritable bowel syndrome, despite its similar name, is a separate, noninflammatory condition). Today roughly 3 in 100,000 people in Hong Kong have a new diagnosis of IBD.^2^ Ng and others call the dramatic increase in IBD cases in Hong Kong alarming.

**Figure:**
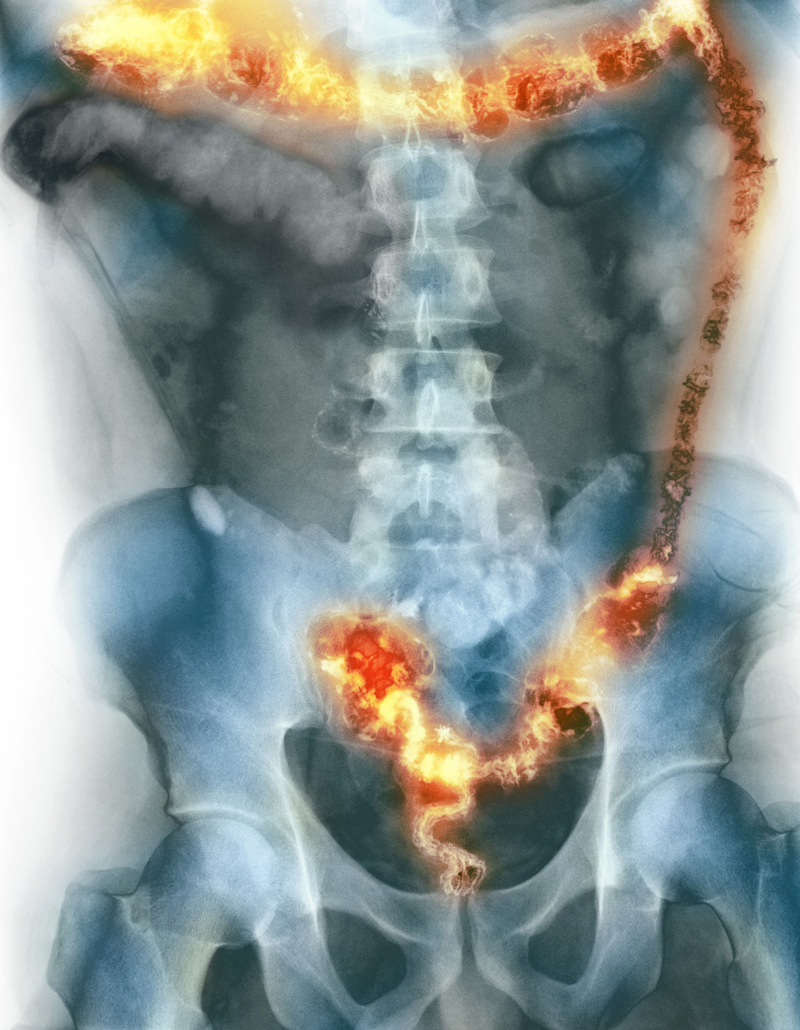
Inflammatory bowel disease (IBD) has become common in the Western world, but its causes remain unclear. With the dramatic increase of cases in Asia in recent years—echoing the disease’s drastic rise in the West decades earlier—investigators have another shot at studying environmental contributors to IBD as it emerges in new populations. © SPL/Science Source

As with many autoimmune diseases, the causes of IBD remain unclear.^3^^,^^4^ Genetic factors, unlike the environment, cannot shift in the span of a single generation, so genes alone can’t explain the recent rise of IBD in non-Western countries. And like many chronic diseases, IBD appears to be the consequence of a complex set of interactions between genes and the environment, an interplay that appears to be modulated by microbes that make their home in the human gut.^5^^,^^6^

Despite recent increases, IBD is still relatively rare in Hong Kong and other parts of Asia compared with North America, Europe, Australia, and New Zealand, where the prevalence of IBD averages about 500 in every 100,000 people in the general population.^1^ The incidence of autoimmune and inflammatory diseases increased drastically in the developed countries of northern Europe and North America during the second half of the twentieth century.^7^ It appeared to be a problem primarily among white people of European descent, but that demographic picture is changing.^7^

“If you look back at the medical literature from a hundred years ago, the rise of IBD—where it was first described in the UK—bears an uncanny resemblance to what we are now witnessing in Hong Kong and across parts of Asia,” says Gil Kaplan, an epidemiologist and gastroenterologist at the University of Calgary who specializes in the global rise of IBD.

Although studies of IBD in Asia are in their infancy, early findings suggest a picture similar to what has been observed in North America. The fingerprints of rapid industrialization—the Westernization of diets, subsequent changes to the gut microbiome, exposure to increased levels of pollution, and even improvements in hygiene and health care—may be linked to IBD risk.^7^^,^^8^ Now Ng and others across Asia and North America are homing in on newly vulnerable populations in search of environmental causes and risk factors—a quest that could ultimately advance efforts to prevent IBD and other chronic inflammatory and autoimmune diseases.

## Inception Cohort

Ng saw her first case of Crohn’s disease in 2010, shortly after she arrived in Hong Kong. The patient, a man in his late thirties, had been diagnosed with abdominal tuberculosis, a disease that is now rare in industrialized countries but still occurs in the developing world. His condition worsened on a standard tuberculosis treatment, and he developed a bowel obstruction. During surgery to unblock his intestine, Ng discovered that the patient had Crohn’s disease.

She began seeing more cases of IBD until soon there were several new patients each month. “We suspected that IBD was becoming more common. We were seeing more cases of it in our center, but we had no data for this trend,” says Ng.

Historically, IBD had been so rare in the Asian population that most physicians suspected some other cause of abdominal pain—an infectious disease or possibly appendicitis.^9^ At the time, some of Ng’s referrals were patients who had suffered gastrointestinal symptoms for years, but whose Crohn’s disease or ulcerative colitis had never been diagnosed. Yet, she thought it seemed unlikely that misdiagnosis alone could account for the rising caseload.

When she looked at the literature, Ng found that nearly all large-scale, population-based studies of IBD had been conducted in Europe, North America, or Australia. No published studies existed to describe Asian populations with IBD—not surprising since IBD in those populations had been virtually nonexistent.^9^ There was no way to compare incidence or prevalence rates among different regions of Asia or between Asia and the rest of the world.^2^

Ng saw an opportunity. In 2011 she initiated the Asia-Pacific Crohn’s and Colitis Epidemiology Study (ACCESS)—a nine-country cohort to determine the incidence of IBD in Asia and to identify potential risk factors.^2^ During a one-year period, Ng helped recruit more than 400 newly diagnosed IBD patients from 21 medical centers for the inception cohort.

Inception cohorts—groups of newly diagnosed patients that allow researchers to study a disease in its earliest stages in a population—are rare and valuable. They offer the unique opportunity to suss out factors involved in an emerging new disease in a population while observing how a society changes over time.^10^ Teasing out those factors can be difficult to do once disease incidence plateaus in a population, as IBD has in much of the Western world, says Kaplan. Armed with decades of disease knowledge and more advanced technology, he says ACCESS represents a second chance to devise a type of epidemiological study that wasn’t possible in the United States or Canada 20 years ago, before IBD incidence began to level off in those countries.

Early findings from ACCESS suggest the highest incidence of IBD lies within highly urbanized regions of Asia, such as Guangzhou, Hong Kong, and Macau.^2^ In regions with more rural inhabitants and less industrialization, IBD incidence remains very low.^2^ Clinical outcomes for IBD, the severity of the disease, and how it presents and progresses in patients are largely similar in Asia when compared with the West, the researchers found.^11^

**Figure:**
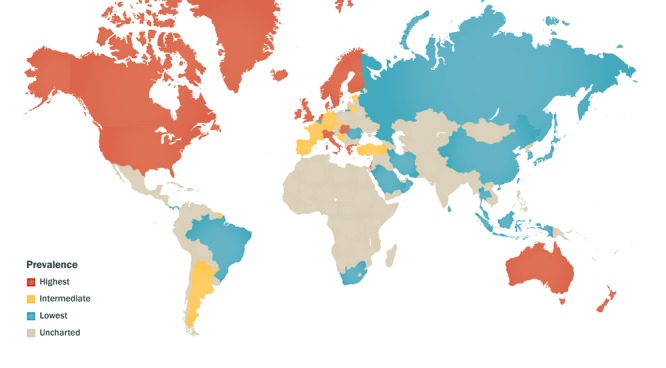
Although rates of IBD in Asia are rising, they are still relatively low compared with industrialized Western areas including North America, several European countries, Australia, and New Zealand. Source: Kaplan (2015)^1^

A complicated picture of environmental risk is also beginning to emerge from the ACCESS cohort.^8^ Ng and colleagues have found that a sedentary lifestyle was associated with an increased risk for Crohn’s disease. Those who breastfed longer as infants and those who had contact with pets during childhood seemed to have a lower risk of IBD.

Surprisingly, findings from ACCESS seem to contradict observations in the Western world that improved sanitation and greater use of antibiotics in childhood can increase the risk of developing IBD—examples of the so-called hygiene hypothesis.^8^ Among the ACCESS cohort, childhood use of antibiotics actually appeared to protect against developing either Crohn’s disease or ulcerative colitis, and people with flush toilets were less likely to have ulcerative colitis.^8^

## Genes + Environment

More than 200 gene regions have been shown to confer risk for Crohn’s disease or ulcerative colitis in people of European descent.^12^ As more data become available on IBD in Asian populations, researchers are finding both similarities and differences in genetic risk factors, compared with Western populations.^7^

The distinct genetic backgrounds in emerging groups, coupled with a lack of replication of certain risk loci previously described in white populations, strengthens the case that environmental exposures play a central role in the development of IBD, according to Ashwin Ananthakrishnan, a gastroenterologist at Massachusetts General Hospital. “Populations where both the incidence and environmental factors are changing simultaneously may offer the best possibility for efficiently identifying risk factors,” he says.

Ontario’s large immigrant population may provide such an opportunity. Canada has among the highest incidence of IBD in the world—about 23.9 cases per 100,000 people per year.^13^ Eric Benchimol, a gastroenterologist at the Children’s Hospital of Eastern Ontario and an epidemiologist at the University of Ottawa and Institute for Clinical Evaluative Sciences, wondered what happens to the incidence of IBD among immigrants who come to Canada from countries where the disease is still quite rare.

Benchimol recently combed through 15 years of health administrative data on all Ontario residents to compare the incidence of IBD between immigrants and non-immigrants.^14^ As expected, immigrants from regions where IBD remains relatively rare—Asia, Latin America, Africa, and the Middle East—had lower rates than native-born Canadians. Incidence of IBD among all immigrants averaged around 7 per 100,000 people per year—far lower than the incidence among non-immigrants.

Yet when Benchimol looked at the Ontario-born children of immigrants, he found something surprising. Kids who were born in Ontario but whose parents were born in South Asia, Africa, and the Middle East had similar risk of IBD as children of non-immigrants; the protective effect of immigration appeared to disappear in the second generation. “Our findings suggest that early-life exposures seem to be a determining factor in predisposing these kids to develop IBD,” says Benchimol.

**Figure:**
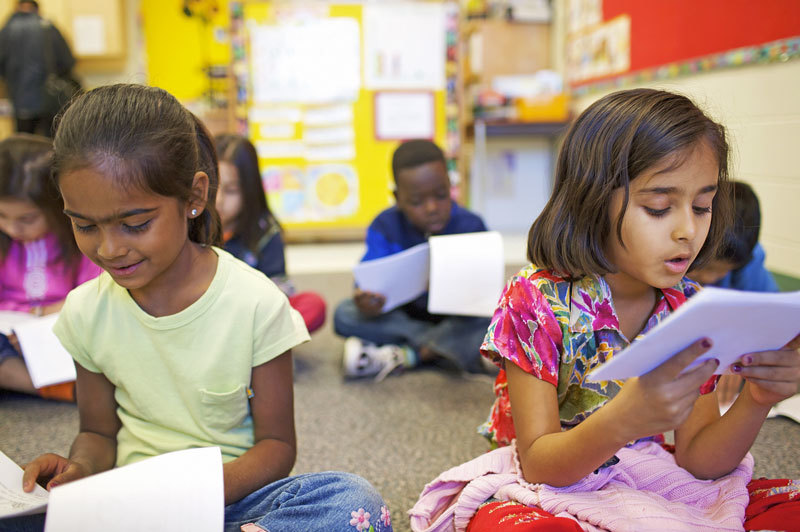
Studies of immigrants in Toronto show that second-generation Canadian children often do not enjoy the same degree of protection against IBD that their parents had in their South Asian home countries. © Ian Taylor/First Light/Corbis

When he looked at asthma, another chronic inflammatory disease that’s on the rise in Asian immigrant populations, he saw a similar pattern.^14^ Compared with non-immigrant children, kids that emigrated from South Asia had much lower rates of asthma. However the Ontario-born children of South Asian immigrants had even higher rates of asthma than the children of non-immigrants. Benchimol and colleagues are working to determine the particular aspects of the Ontario environment and the adoption of a Western lifestyle that can trigger inflammatory diseases in these groups that formerly had enjoyed low risk of disease.

## A Role for the Microbiome

Previous studies have suggested that the gut microbiome may play a role in the development of IBD.^6^ As researchers probe the causes of IBD, they are uncovering a nuanced picture of the complex interactions between genetics, the environment, and the trillions of microorganisms that live in the human gut.^6^ “In IBD, we know that the function of many of the genes that have been linked to disease susceptibility relates to the interplay between the immune system and the microbes in our body,” says Kaplan.

The gut microbiome is the largest reservoir of microbes in the human body.^5^ Commensal gut bacteria perform a number of useful functions—they supply nutrients, help metabolize indigestible compounds, and inhibit the growth of pathogens.^15^

Researchers are now exploring the possibility that certain changes to the gut microbiome can prime an abnormal autoimmune response to these commensal bacteria in genetically susceptible individuals.^5^ Environmental and dietary changes—for instance, exposure to cigarette smoke^16^ and a deficiency in soluble fiber^17^—have been shown in experimental studies to perturb the composition of the gut microbiome and degrade the integrity of the intestinal mucosal barrier.^5^

Sometimes called the body’s second skin, the intestinal mucosal barrier is composed of specialized epithelial cells that help regulate nutrient absorption, gut permeability, and immune homeostasis—functions the immune system performs under steady-state conditions.^18^ Studies in germ-free mice, which are bred in a sterile environment and lack a microbiome, suggest that commensal bacteria can alter gene expression in intestinal epithelial cells.^19^ These findings support a role for the microbiome in regulating immune responses across the mucosal barrier.^5^^,^^18^

To best understand the link between environmental triggers, the gut microbial community, and immune response in humans, “you want to look at healthy people before they get sick and see what is going on in the environment [and] what is changing about the gut microbiome and the immune system as disease develops,” says Ken Croitoru, a gastroenterologist at Mount Sinai Hospital in Ontario. “That’s not an easy experiment to do.”

**Figure:**
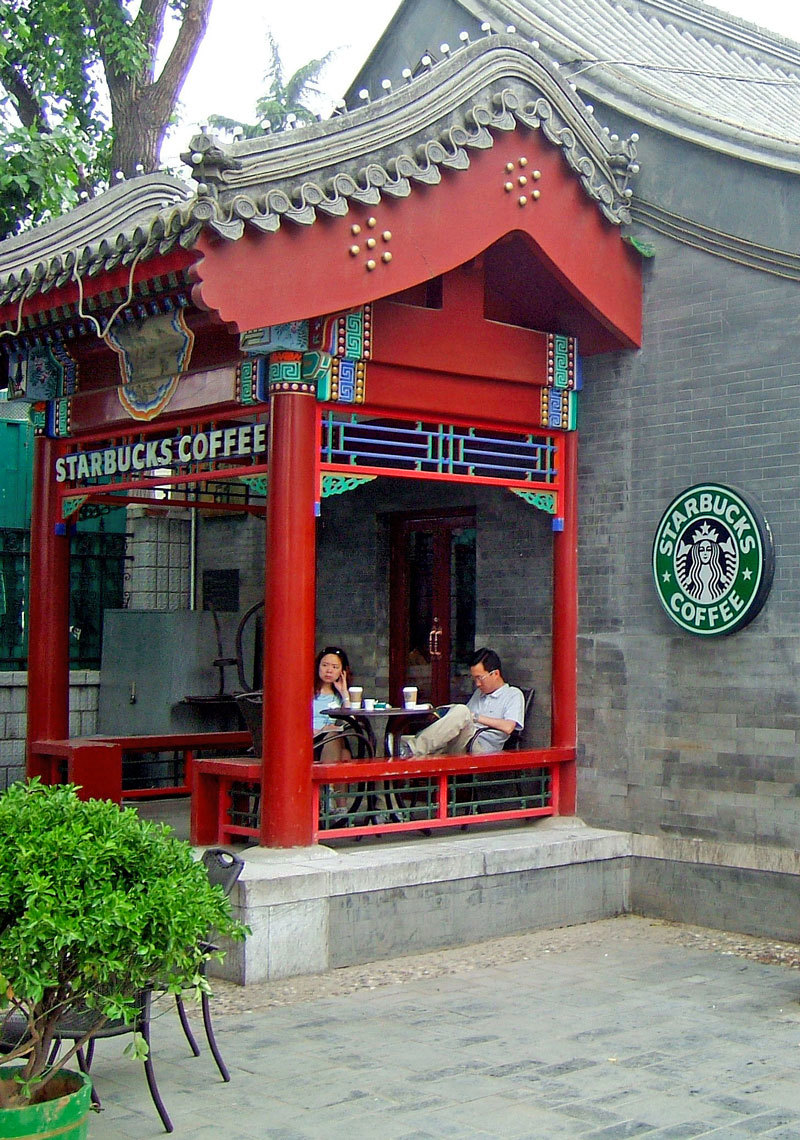
Investigators think the introduction of a Western diet and lifestyle could help explain the increase in IBD in Asia. Diet is a key factor in the makeup of an individual’s gut microbiome, which in turn may play a major role in susceptibility to IBD. © Toby Oxborrow

Croitoru leads the GEM (Genetics, Environmental, Microbial) Project, a prospective cohort of more than 4,000 healthy individuals across Canada, the United States, the United Kingdom, and Israel, which aims to identify factors that contribute to Crohn’s disease.^20^ The study participants all have a sibling or parent with Crohn’s disease, which puts them at higher risk for developing the disease than the general population. Croitoru and colleagues have conducted gut permeability testing to assess the integrity of the intestinal barrier and collected detailed questionnaires on diet and environmental risk factors. Blood samples were used to genotype participants for IBD susceptibility genes, and stool samples enabled the researchers to characterize the composition and diversity of each participant’s gut microbiome.

Since the study started in 2008, 44 study participants have developed Crohn’s disease. Croitoru says the research team will need a total of about 70–75 new diagnoses before they can begin to find any meaningful signals in the data.

Although studies have pointed to a link between a malfunctioning intestinal barrier and IBD, researchers do not yet understand what causes these changes in the intestinal barrier to occur.^5^ People with IBD tend to have a less diverse gut microbial community than healthy individuals, often with a reduction in protective species and an enrichment of potentially harmful bacterial species.^6^ Yet, it’s unclear whether these gut microbial changes cause disease or whether they may be the result of disease processes already taking place in the body.

Jennifer Gommerman, an immunologist at the University of Toronto, is principal investigator of a project to study how the microbiome might change when people migrate from South Asia to Toronto and how those changes could lead to chronic diseases such as IBD.^21^ The project will identify differences between the gut microbial communities of second-generation South Asian Canadians—those identified by Benchimol as being at higher risk of disease—and first-generation immigrants. “It’s a human experiment unfolding before us in the form of migration,” Gommerman says.

## Links to Other Autoimmune Diseases

Researchers and doctors who study and treat a range of autoimmune and chronic inflammatory diseases—including type 1 diabetes, rheumatoid arthritis, and multiple sclerosis (MS)—are looking for clues in the nascent IBD research as well. Like IBD, these diseases appear to be on the rise in Asia’s newly industrialized and transitioning nations.^22^^,^^23^

Many of the gene variants that may increase susceptibility to Crohn’s disease or ulcerative colitis are shared among other immune-mediated diseases, including rheumatoid arthritis and MS, says Kaplan. In addition, studies in rodents have shown that microbial changes in the gut may influence diseases and conditions outside the bowel as well.^5^ “What we think might be happening is that certain people inherit a broad genetic susceptibility to immune-based diseases, and something in the environment then triggers disease,” he says.

But it’s a complicated picture, and it’s not at all clear how environmentally mediated changes to the gut bacteria might change disease risk in genetically susceptible people.^4^ “We know that the microbiome and the immune system are closely linked. The commonality between these various diseases is that the immune system is attacking something it shouldn’t be,” says Gommerman, who also studies the relationship between the immune system and the gut microbiome in MS. Insights gleaned from IBD research about the interplay between the microbiome and the immune system may have broad applicability for other autoimmune and chronic inflammatory diseases, she says.

## Unanswered Questions

It’s not clear just how the changes to the environment wrought by industrialization may have altered the microbiome on a population level.^6^ Experts suspect altered dietary patterns could be playing a big role.^1^ Perhaps one of the most perceptible signs of Westernization across much of Asia in recent years has been the proliferation of fast food restaurants and a widespread adoption of a Western-style diet high in animal protein, processed sugars, starches, and fats, says Ng.

A 2010 study from Italy suggested that diet alone—more than any other variable, studied, including sanitation, hygiene, geography, or ethnicity—may be the dominant factor in shaping the gut microbiome.^7^ The researchers compared the gut microbes of Italian children who consumed a typical Western diet to those of children in the African nation of Burkina Faso, where the diet consists mainly of legumes, grains, and vegetables. They found the Italian children had a much less diverse microbiome than the Burkinabé kids. “The thought is that a more robust microbiome may be better positioned to adapt to environmental insults,” says Kaplan.

A recent study in mice corroborated the findings. Researchers from Stanford University found that a low-fiber diet not only depleted the microbial ecosystem of the mouse gut but that this loss was irreversible, and the microbiome became progressively less diverse over four generations of this diet.^17^ Dietary fiber therefore might play a key role in maintaining a healthy gut microbial community.

Many of the factors that Ng has observed in the ACCESS cohort also suggest impacts of industrialization on the bacterial composition of the gut, which is why the cohort is being expanded to more rural parts of China. The rapid industrialization in some parts of mainland China has created a remarkable contrast between urban and rural areas, she says. Ng believes the answers to what may be spurring the rise of IBD and other autoimmune diseases in Asia could lie in this rural–urban dichotomy.

“We want to go into those rural areas of China where IBD is still really rare,” says Ng. “Compared to people living in China’s megacities, what is different about the environment, about their diets, and their gut microbes?” She hopes that if they can pin down the changes that are most important, they may have a shot at preventing new cases and slowing the global rise of IBD.
